# Beyond ambidexterity: universities and their changing roles in driving regional development in challenging times

**DOI:** 10.1007/s10961-022-09992-4

**Published:** 2023-01-08

**Authors:** Elisa Thomas, Rhiannon Pugh, Danny Soetanto, Sarah L. Jack

**Affiliations:** 1 UiS Business School, University of Stavanger, Stavanger, Norway; 2 CIRCLE – Centre for Innovation Research, Lund University, Lund, Sweden; 3 UniSA Business, University of South Australia, Adelaide, Australia; 4 Stockholm School of Economics, Stockholm, Sweden

**Keywords:** Ambidexterity, Multidexterity, Regional development, University, Innovation, Entrepreneurship

## Abstract

Around the world today, universities are expected to play a unique role as creators of regional growth and innovation. While there appears to be a consensus that the role of universities has been expanded, critiques show that the contribution of universities to their regions is still not well defined. There have been some developments in the literature on the concept of modern universities such as the triple helix, entrepreneurial university and engaged university. However, those concepts focus on enforcing universities’ roles in a single domain such as entrepreneurship, innovation, or civic engagement. Little is known about how universities can facilitate regional growth that goes beyond knowledge transfer activities such as spin-off creation, licensing, and patenting. This paper contributes to a more comprehensive understanding of universities’ role in regional growth through the theoretical lens of ambidexterity. Using ambidexterity, universities with a regional focus were distinguished from those engaged in research commercialization and traditional third-mission roles. Through two case studies, this study found that teaching, research, and engagement should not be separated, since they can serve both economic and social missions. As a result, a new model of multidextrous universities is proposed where universities meet both economic and social missions through teaching, research, and engagement. Contrary to previous contributions which presented universities as ambidextrous organizations where tension appears only between research commercialization and research publication or between teaching and research, this study suggests that universities need to overcome tensions and incorporate a sense of place in all activities to successfully contribute to regional growth.

## Introduction

In this modern knowledge-based economy, universities are required to play a more active role in driving regional development, especially in response to challenging times (Pugh et al., 2016; Thomas & Pugh, 2020). In this context, the entrepreneurial university plays an important role as both a knowledge-producer and a knowledge-disseminator (Guerrero & Urbano, 2012; Gulbrandsen & Slipersaeter, 2007). However, there has been an emerging call for understanding the various roles universities play in their regions, especially looking beyond the most commonly examined economic activities such as spinouts, technology transfer, and commercialisation (Pugh et al., 2018).

Several theories and policy practices have been developed to identify and define the new functions of universities in the region which has created pressure within higher education communities to become more involved in addressing regional societal issues or other contextual problems in the region. As a result, universities are forced to play a ‘complex’ role in their region which has impacted the way in which universities define their missions. Universities in this expanded role need to go beyond their traditional research commercialization role to serve a more holistic regional socioeconomic growth agenda. In order to do this, universities are expected to actively engage in policy development and implementation as well as create stronger relationships with communities and regional partners. The sense of place should be embedded in universities’ mission and integrated into teaching, research, and engagement activities. A critical issue here is that universities are pulled in many directions to serve their region while also acting as an orchestrator for regional socioeconomic growth programmes (Thomas et al., 2020). To meet those objectives, universities require radical changes which pose challenges to universities' management and infrastructure (Cerver Romero et al., 2021).

Despite the importance of the role of universities in regions, theories on universities and policy practices have largely focused on research commercialization and overlooked how regions can benefit from their activities. Additionally, the importance of universities’ developing significant global reputation and impact has forced many universities to develop international collaborations. In doing so, universities run the risk of overlooking the needs of their region and paying less attention to solving local problems. Playing an active role in the region requires universities to shift their paradigm. Universities need to accommodate new objectives and approaches for their teaching and research such as responding to local socioeconomic problems while also actively engaging in contributing to regional growth. With other regional stakeholders, universities should act as drivers for growth and build strong ties with other key players in the region. Having battles on many fronts is what Cerver Romero et al. (2021) call the ‘dual personality’ of universities. As a result, universities should be looking to transform themselves to cope with tensions that arise from these newly defined roles.

This study considers the concept of the ambidextrous organization and asks whether this concept helps us to understand the multiplicity and complexity of the different roles universities are playing within both the economic and social domains in their region. In doing so, this study builds on two already large and vibrant bodies of literature: that of the ambidextrous organization within the management literature, and that pertaining to the concept of universities and regional development. In order to elucidate the simultaneous and overlapping activities contemporary universities are engaged in, the ambidexterity concept has been adapted from management literature into the university context, thus providing a conceptual extension to the existing literature on universities (Audretsch, 2014). However, the ambidexterity concept, with its focus on two conflicting core activities such as exploration and exploitation, we suggest limits us to a binary mode of thinking. Metaphorically, it leads us to think of a person with two hands undertaking different activities at the same time. However, what we find in our empirical research is that the university is more like an octopus with eight arms moving in a sometimes coordinated and sometimes unwieldy manner. As such, we use the concept of ambidexterity to examine conflicts and trade-offs between several different elements of a university to understand the expanding roles of universities in their region, especially how universities adapt their missions in challenging times and cope with management tensions that consequentially arise.

We consider two case studies of universities—one in the United Kingdom and one in Brazil—actively engaged in teaching, research, and the broader social and economic development of their regions, albeit in very different contexts and in different ways. We purposefully chose two contrasting cases, to undertake a wide-ranging consideration of the roles and activities played by contemporary universities in the three missions, i.e., teaching, research, and engagement. The concept of ambidexterity used in this study is derived from a comprehensive literature review that has been operationalized as a framework to analyse the economic and social development initiatives universities are undertaking in their regions. In building our cases, we provide several examples of activities and programmes initiated at the universities to illustrate the range of roles and activities being undertaken in the contemporary university. This leads to a discussion about broadening the context of ambidexterity to multidexterity to account for the multifacetedness inherent in the experiences of these two universities. Multidexterity is, therefore, seen as the key for universities to act in challenging times and to cope with the managerial tensions of promoting regional social and economic development at the same time as providing education and generating new knowledge through research.

## Theoretical framework

### Universities and their region

It has long been appreciated that universities’ roles are evolving, and the latest iteration along this path is that universities are expected to drive innovation and economic development (Pugh et al., 2016, 2019). There has been increasing emphasis on commercializing university research, both as a driver of competitiveness, and on an institutional level to derive financial benefit from research activities (Ambos et al., 2008). Early concepts such as the triple helix, for example, argue that knowledge from universities should be transferred to industry, and then through government to society (Etzkowitz & Leydesdorff, 1997). Although the university plays an important role as a knowledge producer, the success of the model is determined by the interaction of all actors where the university is seen as only one of the main actors in the region.

Another concept used to describe the role universities have played in regional growth is the entrepreneurial university (Guerrero et al, 2014). The concept of the entrepreneurial university has expanded the role of universities to recognize their potential in offering a leading role in an ecosystem and how they act in the creation, application, and exploitation of knowledge to promote economic and social development (Trippl et al., 2015). It is evident that commercialization and outreach, also known as third mission activity, are much more prominent now in the university space and have worked to extend the missions of teaching and research. However, what is striking when we examine the body of work relating to the evolving missions of universities is that there seems to be a greater level of knowledge and understanding around the direct economic activities of universities and their impact when compared to more social or governance-oriented ones. For example, there is an established body of work on spinouts (Clarysse et al., 2005), patents and licenses (Chapple et al., 2005; Sine et al, 2003), and technology transfer (Dolmans et al., 2022; Phongthiya et al., 2022), following the “entrepreneurial university” concept (Guerrero et al, 2014), but much less about the social mission that universities engage in. This finding is critical as the regions require universities to solve both economic and societal problems.

In defining the expanding roles of universities, the concept of the engaged university can be used as a reference. The engaged university concept is an iteration of the “entrepreneurial university” concept (see Audtretsch, 2014), which goes beyond the established perspective of universities’ prominence as a knowledge and innovation driver for economic competitiveness, growth, and wealth creation (Gordon et al., 2012; Guerrero et al., 2015; Larty et al., 2016; Mian, 2011). The idea of the engaged acknowledges the wider roles universities play in influencing its region in a variety of ways such as providing leadership for entrepreneurial thinking and actions (Audretsch, 2014; Chatterton & Goddard, 2000). As universities are expected to do more than just commercialize knowledge in the region, this has the potential to bring about real change in the social space (Maclean et al., 2012).

**Fig. 1:**
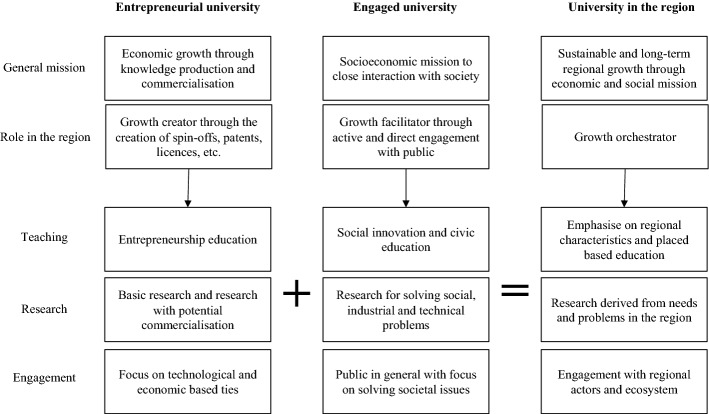
The concept of the university in the region

We argue that the roles of universities in the region can be synthesized from both these concepts, i.e., the entrepreneurial university and the engaged university. Besides pursuing research commercialization and educating students for regional benefits, universities must also be sensitive to regional issues. As a result of these additional expectations, teaching, research, and engagement activities need to adopt a new approach where the region and its actors become inspirations and participating partners. Universities often have to serve national interests and interact with international audiences, which is another challenging task. While adding new objectives with a focus on the region is not easy for universities without a strong regional connection, we see it as a necessary step. Figure 1 illustrates the transformation of concepts.

### Ambidexterity and the new roles of universities

As we explore universities' regional impact, we explore the literature associated with ambidextrous organizations to understand the complexity of evolving multiple roles and activities. Previous studies suggest that ambidexterity is the capability to perform exploration and exploitation simultaneously (Hughes et al., 2007; Kim & Atuahene-Gima, 2010; Lavie & Rosenkopf, 2006; O’Reilly & Tushman, 2004, 2008, 2011). The ambidextrous organization is more likely to achieve superior performance than firms emphasizing one activity at the expense of the others. Exploration involves the pursuit of new areas of activities whereas exploitation covers the use and development of things already known (Levinthal & March, 1993). Exploration entails the development of new knowledge; experimenting to foster the variation and novelty needed for more radical innovation (Atuahene-Gima, 2005); “creativity, experimentation, play and discovery” (Hughes et al, 2007, p. 360). Exploitation can be defined as an extension of current activities; the search for greater efficiency and improvements to enable incremental innovation (Atuahene-Gima, 2005). Maintaining a balance between the two types of activities is a factor in retaining and developing organizational capabilities.

There is a small but interesting body of work examining universities as ambidextrous organizations. Ambidexterity has been used by Ambos et al. (2008) to examine tensions between academic and commercially oriented activities. Moreover, ambidextrous universities have been studied as those jointly developing research publications and research commercialization (Centobelli et al., 2019). In describing such institutions, Chang et al. (2009) discuss that entrepreneurial universities are ambidextrous organizations because they undertake teaching and research, whilst contributing to the economy through the commercialization of technologies, which other researchers discussing “ambidextrous universities” agree on (Beyhan & Findik, 2018; Sengupta & Ray, 2017). In short, these researchers have used the concept of ambidexterity to capture the three missions simultaneously being undertaken by contemporary universities. In this paper, we extend this usage to look specifically at the dual economic and social dimensions of these, as a novel approach to the use of ambidexterity when studying universities in their regions. This contemporaneous role development lends itself to an ambidexterity perspective: existing theory has highlighted the challenge of managing dual or multiple foci (see: Birkinshaw & Gupta, 2013). Indeed, mixed results have been reported when universities try to manage conflicting demands (Markman et al., 2008). Although research has examined the outputs of the effects of these multiple foci, a gap remains when it comes to understanding the root cause of tensions and management issues: the ability to combine conflicting demands (Ambos et al, 2008).

**Fig. 2:**
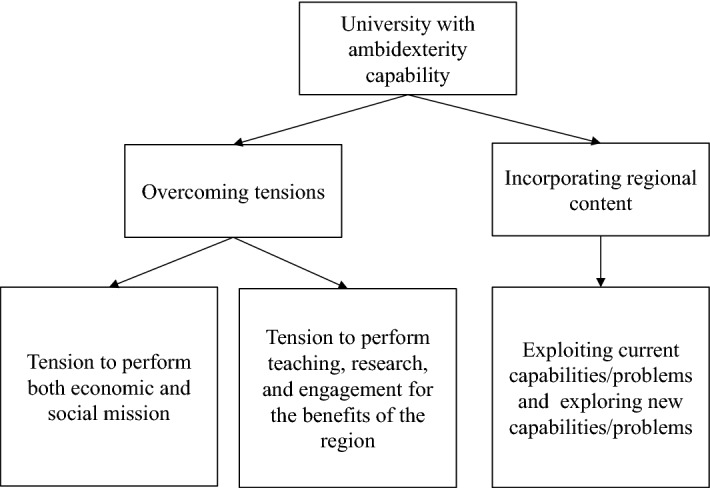
Ambidexterity in the context of universities in the region

In this sense, we perceive a university with ambidexterity capability in two ways, as derived from the literature presented above. Firstly, ambidexterity helps us to envision the different missions or activities conducted simultaneously by universities. We argue that ambidexterity is the solution for overcoming tensions because of performing different missions or activities simultaneously. First, universities need to be ambidextrous to not only pursue commercially oriented activities but to also consider the social mission and regional governance. The second tension refers to the challenges faced by universities in utilizing all their activities for the benefit of the region. Some universities with a lack of ambidexterity may decide to focus on the region via engagement activities. Being ambidextrous means universities use teaching, research, and engagement as a means to make a contribution to their region. Secondly, regional content and the sense of place should be incorporated into the universities’ three missions, i.e., teaching, research, and engagement. We use the concepts of exploration and exploitation to explain how universities continue to pursue their existing and established missions and activities, but also how they add new roles and activities to their work in response to needs at the regional level. The strategic choices of exploitation and exploration are equally important in their quest for defining and redefining their roles in supporting regional growth. This is how we begin our positioning, by considering the sense of place or region as a unique characteristic of the ambidextrous university (Fig. 2).

The next question for this study is how universities achieve ambidexterity. Again, we borrowed the concept from management studies. A view offered by Duncan (1976) suggests accommodating the conflicting tension by shifting organization structures over time to align the organization’s objective and strategy. Here, organizations achieve ambidexterity in a sequential fashion by shifting structure over time. In the context of universities, this option can be done by shifting priority and focus depending on the opportunity. Another solution, offered by Tushman and O’Reilly (1996), proposes organizations explore and exploit in a simultaneous fashion. They suggest this can be accomplished by establishing autonomous exploration and exploitation. In this context, the university may develop a unit focusing on traditional core activities while at the same time creating a unit to explore new opportunities. Another alternative is contextual ambidexterity (Gibson & Birkinshaw, 2004). This approach focuses on the flexibility of individuals to decide how they use their time between exploratory and exploitative activities. From this view, ambidexterity can be achieved by building a system or a culture that encourages individual creativity and control at the same time. Table 1 summarizes the different mechanisms of ambidexterity when one looks at universities’ activities in terms of teaching, research, and engagement.

**Table 1:** Different mechanisms of ambidexterity for universities’ activities

Types of ambidexterity	Overcoming tension in economic and social missions	Overcoming tension in teaching, research, engagement activities
Sequential ambidexterity (Duncan, 1976; Goossen et al., 2012; O’Reilly and Tushman, 2013)	Giving equal opportunities and resources in teaching and research to meet both economic and social objectives	Teaching—Renewing and integrating courses and modules to accommodate new content with a focus on the region Research—Depending on the opportunity, research is conducted Engagement—No structural team is created but the new roles can be undertaken by the established office
Structural ambidexterity (Chang et al., 2009; De Visser et al., 2010; Heracleous et al., 2017; Tushman & O’Reilly, 1996)	Creating special programmes and activities to involve teaching and research as an integral part of universities’ engagement with the region	Teaching—Creating different courses or modules to serve different purposes and pedagogical challenges Research—Maintaining two different streams of research. It is also supported by the creation of a research Center for industrial and policy recommendation Engagement—TTO, Patent office, office for engagement are established to serve additional roles of the university
Contextual ambidexterity (Gibson & Birkinshaw, 2004; McCarthy & Godron, 2011; De Clercq et al., 2014)	Acknowledging the separation of commercialization and social activities in some cases, but at the same time, motivating and encouraging academic staff, students, and graduates to give back to the region	Teaching—Encouraging academics to develop contextual content Research—Allowing more flexibility for academics to direct and manage research activities Engagement—Encouraging academics to participate in regional or local programmes

## Methodological design

In order to develop an understanding of how the concept of the ambidextrous organization explains the multiplicity and complexity of different roles universities are playing, this study follows a qualitative approach through case study research (Yin, 2009). This allowed a more in-depth understanding of the phenomenon of ambidextrous universities and the tensions arising from entrepreneurial universities’ multiple roles. We draw upon two long-term research agendas carried out in two different institutions by researchers formerly working at the institutions selected. The two case studies were carried out separately, but with researchers in common. Both universities have been acting as ambidextrous organizations for decades by combining economic and social missions and have been recognized as examples of good practice in this strategy. Our cases are drawn from (1) Lancaster University (founded 1964) in the United Kingdom where we focussed our research specifically on activities carried out by the management school aimed at regional SMEs, entrepreneurs, and their development; (2) Unisinos (founded 1969) in Brazil where we focus on engagement activities that the university offers to the community and are delivered from different faculties, with the aim of improving the regional economic situation. Unisinos is an established example of a university with a long tradition of helping the region to achieve its economic and social goals (Borba et al., 2005; Grazziotin & Klaus, 2016; Oliveira & Balestrin, 2018; Schmidt et al., 2016; Storck, 2017). In this paper, we select illustrative examples of programmes we have studied that show a range of ways in which universities’ roles and activities have evolved in a complex and multifaceted manner.

A similar study design was used when looking at both cases, drawing on participant observation, document review, and interviews with key stakeholders within these two universities (circa 30 interviews in total). The questions in our interview schedule were organized around topics or themes (Simons, 2009). We conducted “follow-up” interviews in both universities post- Covid-19 to explore how the situation had evolved. In line with procedures used for inductive case study research (Leppäaho et al., 2015), we compiled the information we gathered about each university as a case study. To do this, we pulled together interview transcriptions with the data gathered from documents, field notes and observations. The ideas coming through from the data were looked at against the literature and a constant comparative approach meant that we were able to consider data with the categories and concepts that were emerging (Bansal & Corley, 2012).

Having analysed the programmes which emerged from our interviews as important modes of regional engagement on behalf of the university (Appendix 1), we categorized them according to whether they addressed economic or social missions or both. During the in-depth analysis, we selected three programmes from each institution that we found interesting and that also demonstrated the range of different approaches being taken on both a social and economic level. These programmes were also highlighted by stakeholders we interviewed as important or interesting within the university’s work. The six programmes and activities we analysed illustrate the ambidexterity role of each university in terms of both social and economic missions, and within the established missions of teaching, research, and engagement. Both institutions are large and house several faculties, therefore the task of analysing every single engagement activity across all faculties would have simply been too large for a small research team. As such, there are inevitably activities and roles that we have missed in our analysis.

### Case studies

#### Lancaster: regional governance and network building through integrated activities

Lancaster University is often cited as one of the universities of the UK, with a long tradition of engaging in activities to develop entrepreneurship and business ecosystems. It started in the Northwest of England but increasingly, Lancaster is delivering engagement programmes on a national and international scale. The role of Lancaster University has been well accounted for in previous work (Dada et al., 2016; Pugh et al., 2021), and the institution is seen as quite unique and interesting because of its focus on SMEs and entrepreneurs in the local area, and the sheer volume and scale of support delivered to this group. What is interesting to observe is how the university’s role has increasingly become one of a regional anchor involved in the governance and organization of economic development (Goddard et al., 2014). The university has morphed from delivering commercialization activities and support for businesses to taking on governance roles, in the spirit of the triple helix when the various groups of business, universities, and government, interweave and start taking on each other’s traditional roles (Etzkowitz & Leydesdorff, 1997). To illustrate, we provide three examples of programmes where Lancaster has stepped into governance or regional animateur role going beyond narrow third mission activities. These activities, however, still have an economic underpinning and are oriented toward assisting regional economic development via innovation and entrepreneurship.

At Lancaster University, we analysed three programmes called (1) Wave 2 growth hub programme (W2GH), (2) Entrepreneurs in residence (EIRs), and (3) Innovation, design, entrepreneurship and science (IDEAS), each providing a different aspect of the roles being played by universities, combining both the social and economic elements therein. W2GH is a more social and governance-oriented programme, which works primarily with the public sector to help it set up delivery of small business support for the region. A large programme, receiving 32 million GBP from the central UK government, it worked with organizations across 15 smaller cities in England to set up local growth hubs, the whole programme was managed and coordinated by a core team working within the management school and university support services. The programme was credited with good economic outcomes: over 4000 new jobs were created and around 6000 small businesses were supported. But the intangible results of creating a network of peer support for business support professionals, creating links between the university and central and local government, and embedding interactive learning processes into enterprise policy delivery were arguably just as important.

Entrepreneurs in residence is a very socially oriented programme, linking engagement activities to the teaching mission by bringing local entrepreneurs into the university to deliver teaching and support to entrepreneurship students based on their lived experience. This programme has recently been expanded internationally, so is no longer only focussing on local entrepreneurs, but is a way of linking local entrepreneurs from the Northwest of England into an international network. Finance and funding are not central elements of this programme, it is wholly focussed on sharing knowledge between people and creating links between successful entrepreneurs and the entrepreneurs of the future.

IDEAS is an example (one amongst many) of quite a traditional style of third mission support, that despite the strong leaning towards socially based activities is still important at Lancaster. The IDEAS programme worked with 60 “technology-focused” small and medium-sized enterprises to explore how they could be supported to facilitate growth. Workshops carried out at Daresbury Science and Innovation Campus resulted in 55 jobs being created and 10 being safeguarded. The programme provided business owners with an understanding of their networks, based on research indicating that business growth can be stimulated by optimizing the variety of contacts available to them. The successful application of these concepts, at Daresbury, contributed to the generation of approx. £13.1 m of funding for new regional, national, and international programmes to support a further 2,900 SMEs.

#### Unisinos: social and economic programmes to meet the pressing needs of the region

Unisinos is another documented case of a university with additional socio-economic roles in the region. There is a history of delivering support to the region both in economic terms, but also in social terms. The university stems from a Jesuit network, where the social mission is ingrained in people’s work. Before venturing into the entrepreneurial university path, which has been Unisinos’ strategic investment for the last 20 years, the institution had been involved with regional societal groups who needed help and assistance in basic needs, such as health, nutrition, and youth education. Aligned with public policies for social development, the university has run programmes targeted to children, teenagers, families, and the elderly in vulnerable conditions for more than 40 years. Many of the social programmes also result in economic outputs, and so represent a blended approach to regional engagement. They also feed into the teaching and research activities taking place at the university.

We analyse three different programmes, (1) the Roser Award, (2) help for families in great debt, and (3) laboratory for business reinvention. The Roser Award is a start-up competition run by the start-up incubator, which is part of the technology park on campus, in partnership with Unisinos University since 2012. Although it is very technology-based, it has a clear entrepreneurship focus and the proposals submitted by competing start-ups must help solve local social problems. All competing start-ups receive workshops in subjects related to entrepreneurship, and more than 600 people have been part of the competition since its beginning, with 16 teams participating in 2022. The best projects receive free incubation space and all the services offered by the start-up incubator for some months. The incubatees are primarily university students who want to spin out their ideas. From the winners (from 2012 to 2019), six start-ups are still in the market and with good growth results. The start-up Silo Verde, for example, has patented its technology and is commercializing two connected products: a silo for the storage of grains and a technology platform that monitors and manages the silos. Brazil has a storage deficit of 25% because small farmers cannot afford an adequate storage system. Besides, plastic takes hundreds of years to decompose. The start-up targets these two problems, by producing silos from recycled PET bottles and selling them at a lower price compared to traditional steel silos. Another example is RAKS agricultural technology, which commercializes a low-cost smart system to help small farmers be more profitable by using less water and having better control of soil irrigation. These cases illustrate how the start-ups are solving social and economic needs in tandem and are oriented towards the local needs of the region.

Another Unisinos example is the programme “Help for families in great debt” which delivers free support to families in debt and with a bad credit score. The programme is run in partnership with the local Legal Court of Justice and draws together students and academics from law, social service, and psychology. The aim is to help families renegotiate debt with creditors and delete their names from the national credit bureau’s list of debtors. The programme also offers follow-up sessions and workshops with financial education and financial planning for the future. Feedback from the creditors is that more than 90% of the renegotiations go through. Since 2009, the programme has reached 2608 people and 474 successful agreements.

The third programme we use to exemplify the range of activity at Unisinos is the laboratory for business reinvention. Created in March 2020, because of the negative effects caused by the Covid-19 pandemic on small businesses, Unisinos Business School established an initiative to offer free mentorship in management. In one year, 170 professors and volunteer students delivered mentorship for 112 small and medium enterprises, especially companies depending on the physical business and that suffered a great economic impact. During the Covid-19 lockdown in 2021, the project became a standardized methodology that helped businesses reinvent their activities quickly. Several management issues were addressed urgently in order to allow professors and students to support firms when crises erupted, such as allocating working hours for professors, internally registering the programme to be able to award certificates to students, officialising the programme with the National Registry of Legal Entities and providing physical space on campus.

## Discussion

### Tension and ambidexterity

The empirical work we draw on for this paper is research conducted over the last decade at the two institutions. Whilst we are not presenting a comparative case study, what we do here is bring two different cases together in one paper to conduct a wide-ranging analysis, allowing for deeper theorization and understanding than considering one case alone can elucidate (Stake, 1995; Yin, 2009). Our purpose here is to explore how useful the ambidexterity concept is in helping us to analyse and understand the contemporary roles of universities in their regions in a holistic manner, thus a broad and holistic treatment of the ambidexterity concept (as opposed to a narrow cherry picking from the literature) is required.

From the analysis of the activities conducted by both universities, we found that the ambidexterity concept can be used to explain how the universities conduct multiple, interacting, and competing missions and roles. Whilst a few contributions do exist applying the concept of the ambidextrous organization to the case of universities (Ambos et al, 2008; Beyhan & Findik, 2018; Centobelli et al, 2019; Chang et al, 2009; Sengupta & Ray, 2017), they examine the mix of teaching, research, and commercialization activities, which we see as a limited approach when studying universities’ roles in their regions. We have found that the universities in our study went beyond the traditional three missions and entered the social innovation and governance spheres.

Ambidexterity, and our iteration multidexterity, is certainly context-specific and looks different when we look at institutions with different histories and orientations, in different parts of the world. Located in the countryside but known as a world-class university, Lancaster University is fully embedded in the region through social connections with the local community which trigger the creation of programmes to support local businesses. Similarly, Unisinos’ activities were triggered by its strong ties with local communities. Inspired by the need to solve social economic problems in the region, Unisinos created several business programmes to empower the local economy.

As the nature of ambidexterity is more complex than just balancing an economic or social mission, we propose the concept of multidextrous universities where teaching, research, and engagement activities are meant to support social and economic objectives in the region in addition to allowing the university to solve internal management issues. Multidexterity, what form it takes and how it evolves, is driven by regional context. We argue that multidextrous universities understand the needs and problems facing their regions. In both universities in this study, the missions of the universities evolved based on the regional contexts and needs identified, that the universities recognized they could address. In Lancaster, this was very local SME and entrepreneur focussed, as the main need recognised in the Northwest of England was further support in these areas. At Unisinos, the programmes took a much more poverty and education-oriented angle to the local economy, to meet the high needs of the local population in an unequal society. As such, multidexterity is not a universal concept and evolves in a way that is clearly shaped by the regional context. It is also, by institutional character and history, a fact that has previously been recognised by an organizational focus on ambidexterity within firms (Levinthal & March, 1993; Luger et al., 2018; O'Reilly & Tushman, 2011) which has, nevertheless, largely neglected the regional context which surrounds them. In Unisinos, we see a strong relevance of the Jesuit founding in directing the third mission toward poverty reduction. In Lancaster, the local SME and entrepreneur focus has become ingrained and part of the daily life of the institution.

From the case studies, we also learned that both universities overcome tensions and perform both conflicting objectives and tasks. The examples show that the universities tried to achieve both economic and social objectives and, in some cases, integrated different activities such as teaching or research with engagement activities. Table 2 shows the tensions and how ambidexterity was achieved.

**Table 2:** Case study and the objective of the observation

	Tension and ambidexterity in the context
Lancaster	
Entrepreneurs in residence	Tension was observed in teaching and management. The project shows how ambidexterity is achieved by bringing in local businesses to support the delivery of teaching. It solves the problem of limitation of local knowledge as well as provides place-based education that meets regional/local needs. Students also benefit from receiving a quality education
IDEAS	Tension was observed during engagement and research. The project shows how business engagement was delivered through informed research and how the research was produced as a part of the engagement. While delivering a programme to support local start-ups, data was collected for further research and engagement activities
Wage 2 growth hub (W2GH)	Tension was observed in teaching, research, and engagement. The project was complex and involved all elements of the university. As a result, the university took on the role of manager and orchestrator for a central government-funded regional growth programme
Unisinos	
Roser award	Tension was observed in teaching and engagement: ambidexterity is achieved by combining technology-based solutions brought forward by start-ups to solve local social problems
Help for families in great debt	Tension was observed in research, teaching, and engagement: the programme helps families from the community at the same time serving as internships for law, social service, and psychology students
Laboratory for business reinvention	Tension was observed in research, teaching, and engagement: business engagement was delivered through informed research in the form of mentoring small businesses in the region, at the same time as students from the bachelor’s degree in management learned from being part of mentoring and contact with real business challenges

In previous writings about universities as ambidextrous organizations, the discussion has been mostly focused on adding new roles as exploration and continuing the traditional roles of teaching and research as exploitation. Here we see how the region’s social and economic needs are addressed by university activities, via a process of exploration, to (1) identify needs and opportunities in the region that the university could fulfil, and (2) as exploitation in terms of actually delivering programmes to address these. Interviews with those involved in the long-term with engagement activities illuminated the process of how programmes are designed, implemented, and sometimes ended and new ones are put in their place, and sometimes continued over the long term as part of the university’s regular activities. For some activities, there were longer-term and larger investments, especially things like science parks and incubators, both of which were present in our cases, whereas others were smaller, more piecemeal programmes that might have lasted for shorter periods of time. In this understanding of exploitation and exploration, our findings fundamentally agree with the extant literature (albeit previously researched in the context of firms) in that the central dual tensions of ambidexterity are considered the micro-foundations of the emergence of entrepreneurial opportunities (Busenitz et al., 2003, 2014; Chandrasekaran et al., 2012; Rothaermel & Alexandre, 2009; Vrontis et al., 2017). Whilst this work has found that new ventures have been born or established ventures rejuvenated by these joint processes, we can see that the same can be considered true of programmes and activities implemented by universities. Although these may or may not be profit maximization oriented (for instance, instead, are aimed at the social mission, or regional development outcomes), the centrality of exploration and exploitation might not be so different whether we consider universities or firms. Both Lancaster University and Unisinos have exploited their current capabilities to serve the region but over time, they explore new opportunities and capabilities to expand their roles and contribute more to their region.

### Supporting regional growth in challenging times

While these six programmes are quite different, all address aspects related to a university’s ambidexterity in delivering economic and social roles in its region. In this section of the paper, we illustrate the range of different roles and activities being undertaken. Through the case studies, we also see an evolutionary dynamic taking place whereby the universities are constantly evolving and adding new activities as difficult times arise, such as the Covid-19 business support at UNISINOS, and the regional development programme during the mid-2010s recession in the UK, W2GH. A longitudinal perspective allows us to explore the evolution of missions, and the growing multidexterity that occurs as more missions and dimensions are added to universities’ existing work. Of course, this brings tensions but also possibilities for universities to increase their relevance to their region, as our case studies explore the combination of both social and economic-oriented activities.

In the context of the challenging times Covid-19 brought, it is interesting to consider the evolutionary elements of multidexterity that we have explored and to ask whether these engaged universities are better placed to respond to evolving regional needs in real time. We followed up with our research participants to update our data collected for the Covid-19 era and to find out how both institutions were responding to the current crisis. In both cases, the universities have rapidly developed new programmes and mechanisms to support local businesses struggling in the current scenario, and we suggest that this is in part due to their history of being light-footed and reactive to regional needs through a long-term experience of acting as engaged universities with both social and economic visions. Both institutions have experience in supporting their regional businesses and communities through challenging times. In the UK, it was during the recession and austerity years. In Brazil, it was against the backdrop of economic difficulties and long-term inequalities. We cannot comment decisively on the Covid-19 era, since we do not fully know what the implications on funding, student numbers, academic teaching loads, possible recessions worldwide, and changing third mission landscape will be, placing extra challenges on university management. No doubt, universities will have to act in an even more ambidextrous manner to meet these challenges and to figure out what the problems are going to be in their regions and how they are going to respond to them going forward through their work.

### Multidextrous university: the missing link for university economic and social missions

**Fig. 3:**
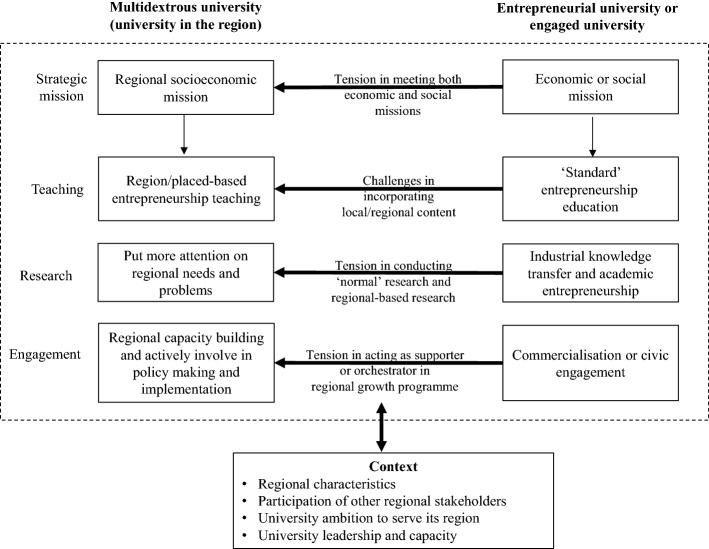
A model of the multidextrous university

To illustrate how multidexterity is manifested at the level of universities’ activities, Fig. 1 shows where the tension emerges as a result of the expanding roles of the entrepreneurial university. It starts from the strategic mission of the university, which can focus on the commercialization of research or civic engagement. However, the multidextrous university needs to merge both the entrepreneurial and engaged university concepts, but with its local context in mind (Fig. 3). Contextual factors, such as regional characteristics, entrepreneurial culture, and the activities of other regional stakeholders, drive and explain the differences multidextrous universities face. For example, Unisinos’ Jesuit configuration supports the emphasis on social outreach programmes, while Lancaster’s long tradition of activities towards entrepreneurship and business supports its pool of programmes.

In terms of teaching, tension arises for the multidextrous university when it needs to move from delivering ‘standard’ entrepreneurial education to developing its own curriculum and programme with a strong sense of place or region. It is expected that the teaching and research conducted by the university meet the characteristics of regional demand. In terms of research, there is a difference between knowledge production to be applied by the industry and knowledge production focused on regional societal needs and problems. For universities with a focus on regional growth, the region and local community can be treated as a source or recipient of knowledge. Knowledge transfer and research commercialization should benefit businesses and communities in the region. Moreover, this new role forces the university to be more flexible and proactive in designing its engagement programme.

## Conclusion: resilience in challenging times

The aim of this study was to develop an understanding of how the concept of the ambidextrous organization explains the multiplicity and complexity of different roles universities are playing. Ambidexterity is a dynamic capability on the basis that universities must reconfigure their competences to maintain a balance between exploring new roles and exploiting current roles in order to adapt to their regional demands. At the same time, universities need to meet both economic and social missions. This is a challenging task as for each activity such as teaching, universities should consider achieving both missions. In terms of research, regional or local issues should get more attention while collaboration with regional or local actors is demanded. The university that behaves ambidextrously likely attempts to meet both economic and social missions in terms of teaching, research, and engagement. Nevertheless, taking these multiple missions has added complexity of issues for university management because it expands their role and activities and takes them away from the main business.

By bringing two existing bodies of work together, ambidextrous organizations and universities in regional development, we propose multidexterity as a more useful and appropriate concept for understanding the various different roles and activities universities play across their three missions. We also bring the perspective of placing universities as organizations within their regions and explore what effect regional dimensions have on how multidexterity evolves and articulates at the organizational level of the university. We found that the context is both a facilitator and a constraint for ambidexterity. The ability of the university to perform ambidextrously will be determined by several contextual factors. For instance, regional characteristics, entrepreneurial culture, and the activities of other regional stakeholders. This expands on previous work on ambidextrous universities (Beyhan & Findik, 2018; Centobelli et al., 2019; Chang et al., 2009; Sengupta & Ray, 2017) which does not address the regional context. We propose multidexterity as both a context-specific and evolutionary concept, and add these two dimensions to the discussion, drawing on the case of two universities we have studied.

In addition to pushing forward our understanding of the role of universities in their regions, we recognized some limitations on the current concept of universities. By employing the ambidexterity concept, and developing it into multidexterity, we can examine the evolution and expansion of the broadly termed “third mission” activities along with the regional component. In this paper, we harness the potential of the multidexterity concept to elucidate this wider range of roles along more-than-economic lines. Because of the scanty focus on universities within the ambidexterity literature and the confusing treatment of the terms therein, and a lack of discussion of regional context in ambidexterity debates more generally, our paper is to a large extent explorative. It draws on two long-term case studies in two very different universities and regions to start to fill in the lacunae in our knowledge about this area. In doing so, this paper pushes forward both the literature derived from the management sphere on organizational ambidexterity and the regional roles of universities from a more economic geography perspective, through combination and cross-fertilization between different fields and ideas. Based on our research question and our particular context, we see that ambidextrous universities use flexibility to manage their activities in an evolutionary dynamic manner, adding new activities whenever difficult circumstances arise (e.g., economic recession, COVID-19, etc.). A more entrepreneurial style of management, one which can deal with uncertainty, and opportunity and has the flexibility to act fast would allow universities to foster regional capacity building. Ambidexterity gives universities the missing link between entrepreneurship, innovation, and management issues, because of the flexibility it offers. As part of this study, we also offer universities another way forward that introduces multidexterity, which allows them to deliver multiple regional roles while dealing with internal challenges.

## Appendix 1

Full list of programmes analysed in each university.

**Table:**

Lancaster	UNISINOS
London creative and digital fusion project	Dance and sports programme
LEAD (business leadership programme)	Elderly networking and bonding
LEAD 2 innovate	Legal practices programme
Berkeley innovation forum	Life with art
KARIM (supporting SME’s international collaboration)	Digital inclusion and citizenship
Lancaster China catalyst programme	Tax and financial services centre
IDEAS	Social-education action in the community
Wave 2 growth hubs	CHANCE (legal services to ex-prisoners)
Evening masterclass	University scholarships to low-income students
GOLD (leadership training)	Cleaner world
Top teams	Help to families in debt
Lancashire and Cumbria BOOST	Roser award
Small business charter	Volunteer mentor
Industrial placements	Small business: all for all
Entrepreneurs in residence	Technological institutes
Campus in the city	Technology transfer office
	Tecnosinos technology park and incubator
